# Special Issue “The Role of Lipids in Health and Diseases”

**DOI:** 10.3390/ijms27114959

**Published:** 2026-05-30

**Authors:** Yutang Wang

**Affiliations:** Discipline of Life Science, Institute of Innovation, Science and Sustainability, Federation University Australia, Ballarat, VIC 3353, Australia; yutang.wang@federation.edu.au

## 1. Introduction

Lipids are hydrophobic biomolecules, typically characterized as waxy or oily and insoluble in water, encompassing a structurally diverse group that includes triglycerides, phospholipids, waxes, and steroids [1,2]. Historically, lipids were regarded primarily as structural components of cellular membranes and as energy storage molecules [3,4]. However, they are now widely recognized as dynamic regulators of a broad range of biological processes, including cellular organization [5,6], energy homeostasis [7,8], and signal transduction [9,10].

Advances in analytical technologies, particularly mass spectrometry-based lipidomics [11,12], have uncovered an extraordinary diversity of lipid species [13,14,15] and elucidated their functional roles in cellular physiology [16,17]. These developments have fundamentally reshaped our understanding of lipids, positioning them as critical molecular determinants of health and disease.

A growing body of evidence demonstrates that the dysregulation of lipid metabolism contributes to a wide spectrum of human diseases [18,19,20]. Aberrant cholesterol handling and lipoprotein metabolism are central to the development of cardiovascular diseases [21,22,23]. Similarly, excessive triglyceride accumulation and lipotoxicity are hallmarks of metabolic disorders, including obesity, type 2 diabetes, and non-alcoholic fatty liver disease (NAFLD) [24,25,26,27,28].

In addition to metabolic diseases, alterations in lipid metabolic pathways play a significant role in cancer pathogenesis. These changes promote tumor cell proliferation [29], enhance migratory capacity [30], support angiogenesis [31], and confer resistance to oxidative stress [32]. Collectively, these findings highlight the critical importance of maintaining lipid homeostasis for overall health [33].

Beyond their roles in metabolism, lipids serve as precursors for a wide range of bioactive mediators that regulate inflammation, immunity [34], and tissue repair [35,36]. Key lipid mediators, including eicosanoids [37], sphingolipids [31], endocannabinoids [38], and specialized pro-resolving mediators [39], orchestrate complex signaling networks that govern both the initiation and resolution of inflammatory responses [40]. Dysregulation of these pathways is implicated in a range of conditions, including inflammatory and autoimmune diseases [34], neurodegenerative disorders [41,42], and cancer [43,44]. Elucidating lipid signaling mechanisms therefore provides critical insights into disease pathogenesis and therapeutic development.

Recent advances in lipid research are increasingly driven by the integration of lipidomics with multi-omics and systems biology approaches, enabling the identification of disease-specific lipid signatures and mechanistic pathways at unprecedented resolution [13,45,46]. Emerging technologies such as precision and spatial lipidomics [47,48], including single-cell lipidomics [49], are revealing cellular heterogeneity and tissue-specific lipid dynamics. These insights are particularly relevant for complex diseases, including cancer [50], cardiovascular disease [51], and neurodegenerative conditions [52].

This Special Issue, The Role of Lipids in Health and Diseases, aims to provide a comprehensive platform for the dissemination of cutting-edge research and critical reviews in lipid biology. By integrating multidisciplinary perspectives, it seeks to advance our understanding of lipid-driven mechanisms and inspire innovative strategies to improve human health.

## 2. Contribution of This Special Issue to the Field of Research

This Special Issue comprises four original research articles [53,54,55,56] and six review papers [57,58,59,60,61,62] (Table 1). Collectively, these ten contributions advance the field of lipid biology by integrating molecular, cellular, and physiological perspectives to elucidate the multifaceted roles of lipids in health and disease.

The articles encompass a broad spectrum of research areas, including mechanistic studies that uncover fundamental aspects of lipid metabolism [55,56,61], membrane fusion [54] and transcriptional regulation, as well as translational investigations linking lipid dysregulation to metabolic [60], neurological [58,59], and genetic disorders [53]. In addition, they explore emerging therapeutic strategies involving lipid modulation, pharmacological interventions, and immunotherapeutic approaches [57].

Together, these contributions underscore the central role of lipids not only as structural components and energy reservoirs but also as critical signaling molecules that regulate diverse biological processes, including glucose homeostasis, neurodegeneration, immune responses, and gut health. These collective insights reinforce the importance of lipid metabolism as a complex and multifunctional target for both fundamental research and clinical innovation.

The topics addressed in this Special Issue are summarized in Table 1, and readers are encouraged to explore the individual articles for detailed insights.

## 3. Concluding Remarks

This Special Issue highlights recent advances in key areas of lipid research, including lipid droplet biology, lipid metabolism, lipid-based immunotherapy, and the impact of lipids on gut health and a range of diseases, such as type 2 diabetes, cancer, and neurological disorders. The integration of multi-omics approaches is expected to further deepen our understanding of lipid functions in health and disease, thereby facilitating the development of personalized medicine strategies.

In parallel, rapid technological progress is accelerating the development of lipid-based therapeutics, particularly lipid nanoparticle platforms for targeted drug delivery [63,64]. Collectively, these advancements reflect a broader shift toward translational and precision lipid research, where mechanistic insights are increasingly harnessed to identify novel biomarkers, therapeutic targets, and tailored interventions aimed at improving clinical outcomes. This is particularly evident in major disease areas such as cardiovascular disease [65,66,67] and cancer [68].

## Figures and Tables

**Table 1:** Summary of the ten articles in this Special Issue.

References	Title	Authors	Main Finding
Original research articles			
[53]	Leptin-Independent Association Between SNVs in the Leptin Gene and HDL-C and Apo-AI in Children	Pomares O, Pérez-Nadador I, Mejorado-Molano FJ et al.	This study shows that specific leptin gene variants are associated with HDL-C and Apo-AI levels in children independently of circulating leptin concentrations.
[54]	Quantifying the Activation Barrier for Phospholipid Monolayer Fusion Governing Lipid Droplet Coalescence	Molotkovsky RJ, Denieva ZG, Senchikhin IN et al.	The authors quantify the energy barrier of phospholipid monolayer fusion, providing mechanistic insight into lipid droplet coalescence processes.
[55]	The Functional Interaction Between PRDM16 and the SREBP Pathway Controls Lipid Metabolism	Mahmood HM, Bengoechea-Alonso MT, Al-Ansari DE et al.	This work demonstrates that PRDM16 interacts functionally with the SREBP pathway to regulate lipid metabolism at the transcriptional level.
[56]	Niemann-Pick C-like Endolysosomal Dysfunction in DHDDS Patient Cells, a Congenital Disorder of Glycosylation, Can Be Treated with Miglustat	Best HL, Cook SR, Waller-Evans H et al.	The study reports that DHDDS-related glycosylation disorders exhibit Niemann-Pick C-like endolysosomal dysfunction, which can be alleviated with Miglustat treatment.
Reviews			
[57]	Invariant Natural Killer T Cells in Cancer Immunotherapy: Lipid-Based Modulation, Nanotechnology, and Translational Advances	Aloliqi AA, Alnuqaydan AM, Alshebremi M et al.	This review highlights how invariant natural killer T cells can be modulated by lipid-based strategies and nanotechnology to enhance cancer immunotherapy.
[58]	The Role of Lipid Alteration in Multiple Sclerosis	Damiza-Detmer A, Pawełczyk M, Głąbiński A	The authors review how lipid alterations contribute to the pathogenesis and progression of multiple sclerosis through effects on inflammation and neurodegeneration.
[59]	St. John’s Wort for Depression: From Neurotransmitters to Membrane Plasticity	Merk VM, Boonen G, Butterweck V	This article proposes that St. John’s Wort exerts antidepressant effects not only via neurotransmitters but also by influencing membrane plasticity and lipid dynamics.
[60]	Triglycerides, Glucose Metabolism, and Type 2 Diabetes	Wang Y	This review explores the interplay between triglyceride metabolism, glucose homeostasis, and the development of type 2 diabetes.
[61]	Crosstalk Between Dietary Fatty Acids and MicroRNAs in the Regulation of Hepatic ApoB-Containing Lipoprotein Synthesis in Humans	Karbowska J, Kochan Z	The study discusses how dietary fatty acids regulate hepatic ApoB-containing lipoprotein production through interactions with microRNAs.
[62]	The Multifaceted Impact of Bioactive Lipids on Gut Health and Disease	Sullivan JP, Jones MK	This review examines how bioactive lipids influence gut health and disease by modulating inflammation, barrier function, and microbiota interactions.

Apo-AI, apolipoprotein-AI; ApoB, apolipoprotein B; DHDDS, dehydrodolichol diphosphate synthetase; HDL-C, high-density lipoprotein cholesterol; PRDM16, PRDI-BF1 and RIZ homology domain containing 16; SNV, single-nucleotide variant; SREBP, sterol regulatory element-binding protein.
